# Adenyl cyclases and cAMP in plant signaling - past and present

**DOI:** 10.1186/1478-811X-8-15

**Published:** 2010-06-25

**Authors:** Chris Gehring

**Affiliations:** 1 Division of Chemistry, Life Science & Engineering, King Abdullah University of Science and Technology, Thuwal 23955-6900, Kingdom of Saudi Arabia

## Abstract

In lower eukaryotes and animals 3'-5'-cyclic adenosine monophosphate (cAMP) and adenyl cyclases (ACs), enzymes that catalyse the formation of cAMP from ATP, have long been established as key components and second messengers in many signaling pathways. In contrast, in plants, both the presence and biological role of cAMP have been a matter of ongoing debate and some controversy. Here we shall focus firstly on the discovery of cellular cAMP in plants and evidence for a role of this second messenger in plant signal transduction. Secondly, we shall review current evidence of plant ACs, analyse aspects of their domain organisations and the biological roles of candidate molecules. In addition, we shall assess different approaches based on search motifs consisting of functionally assigned amino acids in the catalytic centre of annotated and/or experimentally tested nucleotide cyclases that can contribute to the identification of novel candidate molecules with AC activity such as F-box and TIR proteins.

## Is there cAMP-dependent signaling in plants?

By the mid 1970s, 3'-5'-cyclic adenosine monophosphate (cAMP) had been firmly established as an important signaling molecule and second messenger in both animals and lower eukaryotes [1-4]. It was understood that adenyl cyclases (also referred to as adenylyl or adenylate cyclases) (ACs) catalyse the conversion of ATP to cAMP and pyrophosphate, that cAMP affects many different physiological and biochemical processes including the activity of kinases and that cAMP levels decrease in the presence of phosphodiesterases [4]. Given the growing realization of the importance of ACs and cAMP, it is not surprising that plant scientists were keen to learn if this signaling system was universal and therefore operating in plants too. As a result a controversy ensued that is perhaps best summarized by a concluding statement in a review in 1977 - "Our present knowledge, or rather ignorance, of cyclic AMP in higher plants does not permit us to indulge in speculation on its function and thus to increase the disparity between available facts and conclusions, which are based solely on the conviction that plants, with respect to cyclic AMP, should behave like animals or certain bacteria" [5]. Plant scientists were cautioned not to jump to conclusions for two reasons, firstly, the cAMP levels in plants appeared to be low compared to that found in animals and secondly, vagaries of the assays were not conducive to firm conclusions [5]. Whilst more advanced analytical tools have since overcome the second point, the first remains [6]. Reported cAMP levels in plants are typically < 20 pmol/g fresh weight (e.g. ≤12 pmol/g fresh weight in rye grass endosperm cell cultures [7] and < 12 pmol/g fresh weight in *Lilium longiflorum *pistils) whereas animal values are typically > 250 pmol/g wet weight [8]. Incidentally low levels of another cyclic nucleotide, cGMP [9], were also reported in plants where e.g. specific responses to avirulent pathogens increased cytosolic cGMP from < 0.4 pmol/g fresh weight to 1 pmol/g fresh weight. It is however noteworthy that 0.5 pmol/g fresh weight of a cyclic nucleotide corresponds to a cytosolic concentration of approximately 500 pM [6], and that signaling in the picomolar range is not uncommon in plants [10,11]. Despite the low, seemingly un-physiological and certainly not animal-like levels of cAMP in plants, the notion that plants too have a functional cAMP-dependent signal system remained alive, mainly because of the fact that both cell permeant 8-Br-cAMP and stimulation of albeit unknown ACs with forskolin could elicit concentration and time-dependent biological responses such as increases in Ca^2+ ^influx across the plasma membrane [12,13]. Biochemical evidence included the finding that crude alfalfa (*Medicago sativa *L.) root extracts showed calmodulin-dependent AC activity [14].

Arguably the most convincing data for a specific signaling role for cAMP came from whole-cell patch-clamp recordings from *Vicia faba *mesophyll protoplasts that revealed that outward K^+ ^current increased in a dose-dependent fashion following intracellular application of cAMP - and not AMP, cGMP or GMP - and indirect evidence indicated that this modulation occurred through a cAMP-regulated protein kinase [15]. In addition, cAMP-dependent up-regulation of a calcium-permeable conductance activated by hyperpolarization was also reported in guard cells as well as mesophyll cell of *Arabidopsis thaliana *and *Vicia faba *[16].

Cyclic nucleotide gated channels (CNGCs) from olfactory and retinal neurons are members of the S4-containing superfamily of ion channels [17,18] with their unusual stretch of hydrophobic and basic amino acids thought to serve as a transmembrane voltage sensor for channel gating [19]. The first member of this family in plants [20], AKT1, showed homology with both animal cGMP and cAMP gated channels including putative nucleotide binding sites but was described as voltage rather than cyclic nucleotide gated. Subsequently, another member of the Shaker superfamily of K^+ ^channels in Arabidopsis, KAT1, was discovered and also showed the diagnostic cluster of six putative membrane-spanning helices (S1-S6) at the amino terminus of the protein harboring the presumed voltage-sensing region containing Arg/Lys-Xaa-Xaa-Arg/Lys repeats within S4 [21]. This KAT1 was subsequently shown to be a hyperpolarization-activated K^+ ^channel, and importantly, that voltage dependence is also regulated by pH, ATP, and cGMP [22].

In animals systems CNGCs are non-selective cation channels with a role in sensory signal transduction, gated directly by the second messengers cAMP or cGMP. These messengers transduce stimulus-induced changes in their cytosolic concentration into altered membrane potential and cation fluxes as part of the stimulus response cascade. The first such channel characterized in plants was AtCNGC2, a cyclic-nucleotide gated, inward-rectifying K^+ ^channel that is blocked by mmolar concentrations of Ca^2+ ^[23]. AtCNGC2 was later demonstrated to conduct K^+ ^and other monovalent cations but to exclude Na^+ ^and, that membrane transmembrane currents were directly dependent on cAMP [24]. Voltage clamp studies (two-electrode configuration) demonstrated that AtCNGC1 is also a cyclic nucleotide gated channel. In addition, cAMP has been demonstrated to induce inward rectified K^+ ^currents in AtCNGC1 [24]. To date there are >20 Arabidopsis CNGCs annotated and progress has been made in the functional characterisation of several of them, notably AtCNGC2 [25] which plays a key role in innate immunity by facilitating Ca^2+ ^currents and linking them to downstream nitric oxide production critical for the hypersensitive response (HR). Further, AtCNGC4 is permeable to both K^+ ^and Na^+^, is activated by both cGMP and cAMP and is induced in response to pathogen infection and some pathogen-related signals leading to the HR and eventual resistance [26]. Analyses of knock-out lines have revealed that both AtCNGC11 and AtCNGC12 are also positive mediators of resistance against an avirulent biotype of *Hyaloperonospora parasitica *[27]. This may suggest that specific cyclic nucleotide signatures generated in response to biotic [9,28] and abiotic [29] stresses act as messengers in signaling cascades that critically depend on CNGCs [30,31]. Cyclic AMP may also have an important role in abiotic stress responses and in particular responses to NaCl stress since voltage independent channels (VICs) in *Arabidopsis thaliana *roots have been reported to have open probabilities sensitive to μmolar concentrations cAMP or cGMP at the cytoplasmic side [32].

## In search of ACs in higher plants

Given that cAMP plays an important role in signaling in higher plants, it is not surprising that many research groups have put considerable effort into finding ACs and in particular in *Arabidopsis thaliana*. The only annotated and experimentally confirmed AC in plants is a *Zea mays *pollen protein [33] generating cAMP which in turn is a second messenger with a role in polarized pollen tube growth. The Arabidopsis orthologue of this protein (At3g14460) is annotated as disease resistance protein belonging to the nucleotide-binding site-leucine-rich repeat (NBS-LRR) family used for pathogen sensing and with a role in defense responses and apoptosis [34]. NBS-LRR proteins directly bind pathogen proteins and associate with either a modified host protein or a pathogen protein leading to conformational changes in the amino-terminal and LRR domains of NBS-LRR proteins which are thought to promote the exchange of ADP for ATP by the NBS domain. It is thus conceivable that NBS-LRR downstream signaling [34] is enabled by cAMP.

Considering that cyclic nucleotides have important and diverse roles in plant signaling via cyclic nucleotide-responsive protein kinases, -binding proteins and -gated ion channels [35], it is unlikely that a single AC or GC can account for all cAMP and cGMP dependent processes in higher plants. In line with this hypothesis is the fact that a number of Arabidopsis molecules with different domain organizations and experimentally confirmed GC activity have recently been reported [36-38]. It is likely that the same will hold true for ACs and this leaves us with the task to identify them, a task made complicated by the fact that BLAST searches with known and/or experimentally confirmed nucleotide cyclases from lower or higher eukaryotes do not return plausible candidate molecules with low e-values. We also note that Prosite signatures for class one and two ACs ((EYFG[SA]X(2)LWXLYK) and (YRNXW[NS]E[LIVM]RTLHFXG)) are not present in the Arabidopsis proteome even if we allow 2 mismatches.

**Figure 1:**
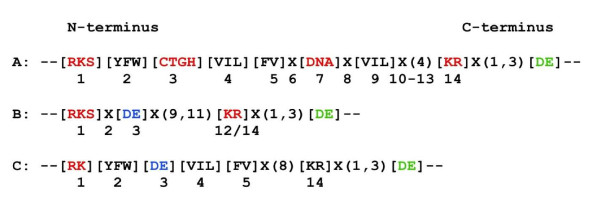
**Catalytic centre motifs of nucleotide cyclases**. (A) Centre motif of experimentally tested GCs in plants. The residue (red) in position 1 does the hydrogen bonding with the guanine, the amino acid in position 3 confers substrate specificity and the residue in position 14 stabilises the transition (GTP/cGMP). The Mg^2+^/Mn^2+^-binding site is C-terminal (green). In the derived motifs (B and C) specific for ACs, position 3 (blue) has been substituted to [DE] to allow for ATP binding.

In Arabidopsis, functionally tested GCs have been identified with a 14 amino acid long search term [36] deduced from an alignment of conserved and functionally assigned amino acids (Figure 1A) in the catalytic centre of annotated GCs [39,40] from lower and higher eukaryotes. For this reason we may expect that a similar approach might lead to the discovery of novel ACs. In the modified AC search terms the amino acid residues that confer substrate specificity (position 3 in Figure 1) will be substituted to [DE]. Consequently, the core motif within the catalytic centre consists of the functionally assigned residue that does the hydrogen bonding with the adenine (position 1), the amino acid that confers substrate specificity for ATP (position 3) and the amino acid that stabilizes the transition state from ATP to cAMP ([K,R], position 12-14). Additional diagnostic residues are the Mg^2+^/Mn^2+ ^binding amino acid [E,D], usually 1 - 3 amino acids removed from C-terminal of the transition state stabilizing residue. Such a modified pattern ([RKS]X[DE]X(9,11)[KR]X(1,3)[ED]) (Figure 1B) is present in a *Zea mays *AC (AJ307886.1) which is the only experimentally tested AC in plants and it is also present in the *Sorghum bicolor *ortholog (gb|EER90437.1) and the related (2e^-70^) Arabidopsis NBS-LRR class protein (At3g14460).

In many GCs and all experimentally confirmed GCs in Arabidopsis [36-38], the position between the assigned residue that does the hydrogen bonding (position 1) and the amino acid that confers substrate specificity (position 3) of the core motif is [YFW] and this is also the case in the confirmed *Zea mays *AC which incidentally shares some similarity (5e^-05^) with an annotated *Neurospora crassa *AC (XP_965280.1).

In Arabidopsis (TAIR: http://www.arabidopsis.org) there are currently 3 annotated but functionally unconfirmed ACs (At1g26190, At1g73980 and At2g11890) and they all do contain the motif [RKS]X[DE]X(9,11)[KR]X(1,3)[DE] (Figure 1B). The first, a phosphoribulokinase/uridine kinase family protein shows similarity (3e^-108^) to an AC domain-containing protein from *Polysphondylium pallidum *PN500. This protein contains a conserved CYTH-like domain typical for the superfamily of enzymes that hydrolyze triphosphate-containing substrates and require metal cations as cofactors. The term CYTH derives from bacterial class IV adenylyl cyclases (CyaB) and thiamine triphosphatase and the domain occurs in RNA triphosphatases, membrane-associated polyphosphate polymerases, tripolyphosphatases, nucleoside triphosphatases, nucleoside tetraphosphatases and other proteins with unknown functions. Furthermore, searches initiated from the C-terminal region to the uridine kinase from *Oryza sativa *identified archaeal CyaB homologs [41]. The second candidate AC, At1g73980 has a similar domain organisation and high homology (6e^-109^) to a *Dictyostelium discoideum *AX4 AC domain containing protein. The third has only some similarity to non plant proteins, one being a *Trichomonas vaginalis *G3 AC family protein (2e^-04^). In addition, there is one putative Arabidopsis AC (At3g21465) annotated at NCBI http://www.ncbi.nlm.nih.gov/protein/51968402. It does contain the core motif, but has no annotated domains or known functions and does not share any similarity with annotated and/or experimentally confirmed ACs but appears to be transcriptionally up-regulated in response to biotic stress.

While the presence of the core motif may prove useful as supporting criterion for the identification of candidate ACs, it is not stringent enough to identify candidate ACs *ab initio*. In order to achieve this, I propose to use a previously identified 14 amino acid long GC catalytic centre search motif modified for specificity for ATP rather than GTP binding and with the C-terminal metal binding residue ([RK][YFW][DE][VIL][FV]X(8)[KR]X(1,3)[DE]) (Figure 1C). This motif retrieves 9 candidate Arabidopsis ACs (Table 1) including 2 F-box proteins (At3g28223 and At4g39756) and a toll interleukin receptor nucleotide-binding site leucine rich repeat protein (TIR-NBS-LRR; At3g04220). In the former, the F-box domains (cyclin like; IPR001810) have a role in protein-protein interactions and have also been associated with cellular functions such as signal transduction and the regulation of the cell cycle that in turn is linked to both auxin responses and changes in cellular cAMP content [42-44]. Given this association one might be tempted to speculate that *axi*, an auxin independence conferring gene could encode an AC, particularly since the Arabidopis axi1 protein does contain the AC core motif as well as the C-terminal metal binding residue.

**Table 1:** *Arabidopsis thaliana *proteins containing the AC search motif: [RK][YFW][DE][VIL][FV]X(8)[KR]X(1,3)[DE]

**ATG No.**	**Sequence**	**Annotation**
At1g25240	-KWEIFEDDFCFTCKDIKE-	Epsin N-terminal homology
At1g62590	-KFDVVISLGEKMQR--LE-	Pentatricopeptide (PPR) prot.
At1g68110	-KWEIFEDDYRCFDR--KD-	Epsin N-terminal homology
At2g34780	-KFEIVRARNEELKK-EME-	MATERNAL EFFECT EMBRYO ARREST 22
At3g02930	-KFEVVEAGIEAVQR--KE-	Chloroplast protein
At3g04220	-KYDVFPSFRGEDVR--KD-	TIR-NBS-LRR class
At3g18035	-KFDIFQEKVKEIVKVLKD-	Linker histone-like prot. - HNO4
At3g28223	-KWEIVSEISPACIKSGLD-	F-box protein
At4g39756	-KWDVVASSFMIERK--CE-	F-box protein

With regards to At3g04220, we note that LRR proteins with AC domains have been reported [45]. Further, the *Zea mays *AC [33] is structurally similar to plant TIR-NBS-LRR type disease resistance proteins (e.g. ADB66335.1, *Populus trichocarpa *4e^-76^) and At3g04220 also contains a P-loop NTase signature which also occurs in signal transduction ATPases with numerous domains (STAND) that in turn include ACs [46].

## Conclusion and outlook

Given that our understanding of the structural features that enable nucleotide cyclase activity in higher plants is growing, we can expect significant progress in the discovery and experimental confirmation of novel ACs in higher plants in the near future. Consequently, this will afford a better understanding of the role of cAMP as second messenger in plant development, responses to environmental stimuli and/or hormones. In addition, we are likely to see cAMP-dependent transcriptomes and (phospho-)proteomes, that, together with studies in mutants, will afford new and fundamental insights into plant signaling.

## Competing interests

The author declares that they have no competing interests.

## References

[B1] Goodman DB Rasmussen H DiBella F Guthrow CE Jr Cyclic adenosine 3':5'-monophosphate-stimulated phosphorylation of isolated neurotubule subunits Proc Natl Acad Sci USA 1970 67 652 659 10.1073/pnas.67.2.652 4331718 PMC283255

[B2] Gerisch G Hulser D Malchow D Wick U Cell communication by periodic cyclic-AMP pulses Philos Trans R Soc Lond B Biol Sci 1975 272 181 192 10.1098/rstb.1975.0080 1814

[B3] Wiegant VM Cyclic nucleotides in nervous tissue Brain Res Bull 1978 3 611 622 10.1016/0361-9230(78)90007-2 233772

[B4] Robison GA Butcher RW Sutherland EW Cyclic AMP Annu Rev Biochem 1968 37 149 174 10.1146/annurev.bi.37.070168.001053 4299844

[B5] Amrhein N The current status of cyclic AMP in higher plants Ann Rev Plant Physiol 1977 28 123 132 10.1146/annurev.pp.28.060177.001011

[B6] Assmann SM Cyclic AMP as a second messenger in higher Plants (Status and future prospects) Plant Physiol 1995 108 885 889 12228514 10.1104/pp.108.3.885 PMC157436

[B7] Ashton AR Polya GM Cyclic adenosine 3':5'-monophosphate in axenic rye grass endosperm cell cultures Plant Physiol 1978 61 718 722 10.1104/pp.61.5.718 16660372 PMC1091964

[B8] Butcher RW Baird CE Sutherland EW Effects of lipolytic and antilipolytic substances on adenosine 3',5'-monophosphate levels in isolated fat cells J Biol Chem 1968 243 1705 1712 4384670

[B9] Meier S Madeo L Ederli L Donaldson L Pasqualini S Gehring C Deciphering cGMP signatures and cGMP-dependent pathways in plant defence Plant Signal Behav 2009 4 307 309 10.4161/psb.4.4.8066 19794847 PMC2664491

[B10] Ehrhardt DW Atkinson EM Long SR Depolarization of alfalfa root hair membrane potential by *Rhizobium meliloti *nod factors Science 1992 256 998 1000 10.1126/science.10744524 10744524

[B11] Morse M Pironcheva G Gehring C AtPNP-A is a systemically mobile natriuretic peptide immunoanalogue with a role in *Arabidopsis thaliana *cell volume regulation FEBS Lett 2004 556 99 103 10.1016/S0014-5793(03)01384-X 14706834

[B12] Kurosaki F Nishi A Stimulation of calcium influx and calcium cascade by cyclic AMP in cultured carrot cells Arch Biochem Biophys 1993 302 144 151 10.1006/abbi.1993.1192 8385897

[B13] Kurosaki F Kaburaki H Nishi A Synthesis and degradation of cyclic AMP in cultured carrot cells treated with forskolin Arch Biochem Biophys 1993 303 177 179 10.1006/abbi.1993.1270 8387751

[B14] Carricarte VC Bianchini GM Muschietti JP Tellez-Inon MT Perticari A Torres N Flawia MM Adenylate cyclase activity in a higher plant, alfalfa (*Medicago sativa*) Biochem J 1988 249 807 811 3128270 10.1042/bj2490807 PMC1148778

[B15] Li W Luan S Schreiber SL Assmann SM Cyclic AMP stimulates K^+ ^channel activity in mesophyll cells of *Vicia faba *L Plant Physiol 1994 106 957 961 10.1104/pp.106.3.957 7529928 PMC159618

[B16] Lemtiri-Chlieh F Berkowitz GA Cyclic adenosine monophosphate regulates calcium channels in the plasma membrane of Arabidopsis leaf guard and mesophyll cells J Biol Chem 2004 279 35306 35312 10.1074/jbc.M400311200 15199067

[B17] Heginbotham L Abramson T MacKinnon R A functional connection between the pores of distantly related ion channels as revealed by mutant K^+ ^channels Science 1992 258 1152 1155 10.1126/science.1279807 1279807

[B18] Jan LY Jan YN A superfamily of ion channels Nature 1990 345 672 10.1038/345672a0 1694264

[B19] Stuhmer W Conti F Suzuki H Wang XD Noda M Yahagi N Kubo H Numa S Structural parts involved in activation and inactivation of the sodium channel Nature 1989 339 597 603 10.1038/339597a0 2543931

[B20] Sentenac H Bonneaud N Minet M Lacroute F Salmon JM Gaymard F Grignon C Cloning and expression in yeast of a plant potassium ion transport system Science 1992 256 663 665 10.1126/science.1585180 1585180

[B21] Anderson JA Huprikar SS Kochian LV Lucas WJ Gaber RF Functional expression of a probable *Arabidopsis thaliana *potassium channel in Saccharomyces cerevisiae Proc Natl Acad Sci USA 1992 89 3736 3740 10.1073/pnas.89.9.3736 1570292 PMC525565

[B22] Hoshi T Regulation of voltage dependence of the KAT1 channel by intracellular factors J Gen Physiol 1995 105 309 328 10.1085/jgp.105.3.309 7769379 PMC2216946

[B23] Leng Q Mercier RW Yao W Berkowitz GA Cloning and first functional characterization of a plant cyclic nucleotide-gated cation channel Plant Physiol 1999 121 753 761 10.1104/pp.121.3.753 10557223 PMC59437

[B24] Leng Q Mercier RW Hua BG Fromm H Berkowitz GA Electrophysiological analysis of cloned cyclic nucleotide-gated ion channels Plant Physiol 2002 128 400 410 10.1104/pp.010832 11842144 PMC148903

[B25] Ali R Ma W Lemtiri-Chlieh F Tsaltas D Leng Q von Bodman S Berkowitz GA Death don't have no mercy and neither does calcium: Arabidopsis CYCLIC NUCLEOTIDE GATED CHANNEL2 and innate immunity Plant Cell 2007 19 1081 1095 10.1105/tpc.106.045096 17384171 PMC1867353

[B26] Balague C Lin B Alcon C Flottes G Malmstrom S Kohler C Neuhaus G Pelletier G Gaymard F Roby D HLM1, an essential signaling component in the hypersensitive response, is a member of the cyclic nucleotide-gated channel ion channel family Plant Cell 2003 15 365 379 10.1105/tpc.006999 12566578 PMC141207

[B27] Yoshioka K Moeder W Kang HG Kachroo P Masmoudi K Berkowitz G Klessig DF The chimeric Arabidopsis CYCLIC NUCLEOTIDE-GATED ION CHANNEL11/12 activates multiple pathogen resistance responses Plant Cell 2006 18 747 763 10.1105/tpc.105.038786 16461580 PMC1383647

[B28] Ma W Yoshioka K Gehring C Berkowitz GA Demidchik V, Maathuis F The function of cyclic nucleotide-gated channels in biotic stress Ion Channels and Plant Stress Responses, Signaling and Communication in Plants Springer-Verlag Berlin Heidelberg (Germany)

[B29] Donaldson L Ludidi N Knight MR Gehring C Denby K Salt and osmotic stress cause rapid increases in *Arabidopsis thaliana *cGMP levels FEBS Lett 2004 569 317 320 10.1016/j.febslet.2004.06.016 15225654

[B30] Kaplan B Sherman T Fromm H Cyclic nucleotide-gated channels in plants FEBS Lett 2007 581 2237 2246 10.1016/j.febslet.2007.02.017 17321525

[B31] Talke IN Blaudez D Maathuis FJ Sanders D CNGCs: prime targets of plant cyclic nucleotide signalling? Trends Plant Sci 2003 8 286 293 10.1016/S1360-1385(03)00099-2 12818663

[B32] Maathuis FJ Sanders D Sodium uptake in Arabidopsis roots is regulated by cyclic nucleotides Plant Physiol 2001 127 1617 1625 10.1104/pp.010502 11743106 PMC133566

[B33] Moutinho A Hussey PJ Trewavas AJ Malho R cAMP acts as a second messenger in pollen tube growth and reorientation Proc Natl Acad Sci USA 2001 98 10481 10486 10.1073/pnas.171104598 11517303 PMC56986

[B34] DeYoung BJ Innes RW Plant NBS-LRR proteins in pathogen sensing and host defense Nat Immunol 2006 7 1243 1249 10.1038/ni1410 17110940 PMC1973153

[B35] Newton RP Smith CJ Cyclic nucleotides Phytochemistry 2004 65 2423 2437 10.1016/j.phytochem.2004.07.026 15381406

[B36] Ludidi N Gehring C Identification of a novel protein with guanylyl cyclase activity in *Arabidopsis thaliana* J Biol Chem 2003 278 6490 6494 10.1074/jbc.M210983200 12482758

[B37] Kwezi L Meier S Mungur L Ruzvidzo O Irving H Gehring C The *Arabidopsis thaliana *brassinosteroid receptor (AtBRI1) contains a domain that functions as a guanylyl cyclase *in vitro* PLoS One 2007 2 e449 10.1371/journal.pone.0000449 17520012 PMC1867859

[B38] Meier S Ruzvidzo O Morse M Donaldson L Kwezi L Gehring C The Arabidopsis wall associated kinase-like 10 gene encodes a functional guanylyl cyclase and is co-expressed with pathogen defense related genes PLoS One 2010 5 e8904 10.1371/journal.pone.0008904 20126659 PMC2811198

[B39] Liu Y Ruoho AE Rao VD Hurley JH Catalytic mechanism of the adenylyl and guanylyl cyclases: modeling and mutational analysis Proc Natl Acad Sci USA 1997 94 13414 13419 10.1073/pnas.94.25.13414 9391039 PMC28319

[B40] McCue LA McDonough KA Lawrence CE Functional classification of cNMP-binding proteins and nucleotide cyclases with implications for novel regulatory pathways in *Mycobacterium tuberculosis* Genome Res 2000 10 204 219 10.1101/gr.10.2.204 10673278

[B41] Iyer LM Aravind L The catalytic domains of thiamine triphosphatase and CyaB-like adenylyl cyclase define a novel superfamily of domains that bind organic phosphates BMC Genomics 2002 3 33 10.1186/1471-2164-3-33 12456267 PMC138802

[B42] Ehsan H Reichheld JP Roef L Witters E Lardon F Van Bockstaele D Van Montagu M Inze D Van Onckelen H Effect of indomethacin on cell cycle dependent cyclic AMP fluxes in tobacco BY-2 cells FEBS Lett 1998 422 165 169 10.1016/S0014-5793(97)01610-4 9489998

[B43] Leyser O Auxin signalling: protein stability as a versatile control target Curr Biol 1998 8 R305 307 10.1016/S0960-9822(98)70193-9 9560337

[B44] Mohanty S Lee S Yadava N Dealy MJ Johnson RS Firtel RA Regulated protein degradation controls PKA function and cell-type differentiation in Dictyostelium Genes Dev 2001 15 1435 1448 10.1101/gad.871101 11390363 PMC312710

[B45] Suzuki N Choe HR Nishida Y Yamawaki-Kataoka Y Ohnishi S Tamaoki T Kataoka T Leucine-rich repeats and carboxyl terminus are required for interaction of yeast adenylate cyclase with RAS proteins Proc Natl Acad Sci USA 1990 87 8711 8715 10.1073/pnas.87.22.8711 2247439 PMC55029

[B46] Leipe DD Koonin EV Aravind L STAND, a class of P-loop NTPases including animal and plant regulators of programmed cell death: multiple, complex domain architectures, unusual phyletic patterns, and evolution by horizontal gene transfer J Mol Biol 2004 343 1 28 10.1016/j.jmb.2004.08.023 15381417

